# Innate-Adaptive Immune Crosstalk 2016

**DOI:** 10.1155/2017/3503207

**Published:** 2017-04-11

**Authors:** Anil Shanker, Menaka C. Thounaojam, Manoj K. Mishra, Mikhail M. Dikov

**Affiliations:** ^1^Department of Biochemistry and Cancer Biology, School of Medicine, Meharry Medical College, Nashville, TN 37208, USA; ^2^Host-Tumor Interactions Research Program, Vanderbilt-Ingram Comprehensive Cancer Center, Vanderbilt University, Nashville, TN 37232, USA; ^3^Vanderbilt Center for Immunobiology, Vanderbilt University, Nashville, TN 37232, USA; ^4^Vanderbilt Center for Translational and Clinical Immunology, Vanderbilt University, Nashville, TN 37232, USA; ^5^Department of Ophthalmology, Medical College of Georgia, Augusta University, Augusta, GA 30912, USA; ^6^Cancer Biology Research and Training Program, Department of Biological Sciences, Alabama State University, Montgomery, AL 36104, USA; ^7^Department of Medicine, James Cancer Center, The Ohio State University, Columbus, OH 43210, USA

The vertebrate immunological defense system relies upon interdependent regulatory interactions between the innate and adaptive immune compartments playing a pivotal role in various physiological and immunopathological conditions. It is, thus, imperative to study both of these immune compartments as one functional unit. Detailed understanding of the interaction between innate and adaptive immunity can lead to new approaches aimed to improve immunotherapy for various diseases. After receiving an overwhelming number of submissions and successful compilation of research/review articles on the innate-adaptive immune crosstalk in the first season, this journal made a decision to publish an annual special issue on this subject. This 2016 special issue consists of 10 review and 6 research articles accepted from a total of 25 submissions following a rigorous peer review.

This special issue includes three interesting review articles on the importance of NK cells in innate and adaptive immune functions. A review by S. Gabrielli et al. discussed the importance of NK cell memories and the innate-adaptive immune intrinsic crosstalk. Another article by R. Thomas and X. Yang reviewed literature on NK-DC crosstalk in immunity to microbial infections with special emphasis on the ability of various microbes to differentially influence NK-DC crosstalk. J.-Q. Yang et al. discussed effects of invariant NKT cells on parasitic infections and hygiene hypothesis. They highlighted relevance of the natural ligands from parasites and the involvement of iNKT cells in the hygiene hypothesis. Lipid-specific T cells comprise a group of T cells that recognize lipids bound to the MHC class I-like CD1 molecules. In a review by C. S. Pereira and M. F. Macedo, the characteristics of CD1 molecules and CD1-restricted lipid-specific T cells were described to highlight the innate-like and adaptive-like features of different CD1-restricted T cell subtypes. Osteopontin (OPN) regulates the immune response at multiple levels. The review by N. Clemente et al. described the OPN structure, isoforms, and functions and its role in regulating the crosstalk between innate and adaptive immunity in autoimmune diseases. Deficiency of Zinc (Zn), an essential micronutrient, has been shown to suppress both innate and adaptive immune responses. In this review, S. Hojyo and T. Fukada highlighted the emerging functional roles of Zn and Zn transporters in immunity, focusing on how the crosstalk between Zn and immune-related signaling guides the normal development and function of immune cells. In a review article entitled “Crosstalk between innate lymphoid cells and other immune cells in the tumor microenvironment”, F. Flores-Borja et al. defined the specific contributions of each type of innate lymphoid cells leading to tumor development and discussed how the manipulation of their function will be important to develop new anticancer interventions and therapeutic strategies. In a review article on advances in immunotherapy for glioblastoma multiforme (GBM), B. Huang et al. compiled studies on the use of immunotherapy for GBM and discussed their lacunae. Further, K. Zhou et al. discussed recent advances of the NLRP3 inflammasome in central nervous system disorders. The review by C. Zhang et al. summarized recent research studies in the field of MDSCs and the role of immune cell crosstalk in the establishment and maintenance of immune tolerant microenvironment in transplantation and offered future research directions.

The research articles published in this annual special issue include an interesting article on therapeutic modulation of innate and adaptive immune responses. L. Tan et al. reported efficacy of dietary supplementation of *Astragalus* polysaccharides to enhance immune components and growth factors EGF and IGF-1 in sow colostrum (the main external resource providing piglets with nutrients and maternal immune molecules). In another article, S. Gorman et al. showed that dietary supplementation of vitamin D increased the percentages and suppressive activity of cells in the skin-draining lymph nodes, which are poised to suppress dermal inflammation. This special issue also contains two articles on immune modulation during bacterial infections. G. F. Schiavano et al. evaluated changes in STAT-1 activation after the uptake of two Gram-positive and two Gram-negative bacteria in human macrophages. Authors concluded that STAT-1 activation is independent of the internalized bacterial number and IFN-*γ* release. The other articles in this special issue focused on preclinical and clinical cancer immunology aspects. S. McNeal et al. evaluated association of immunosuppression with death receptor-6 expression during the development and progression of spontaneous ovarian cancer in laying hen model. In the other clinical study, 13 patients were treated with metastatic or locally advanced pancreatic cancer using autologous RetroNectin-activated CIK cells (R-CIK cells) alone or in combination with chemotherapy. Authors concluded that RetroNectin might enhance antitumor activity of CIK cells and it is safe for use in treating pancreatic cancer. Finally, an article by R. H. M. Raeven et al. performed a meta-analysis of pulmonary transcriptomes from differently primed mice to identify molecular signatures of differentiation leading to immune responses following *Bordetella pertussis* infection.

